# A T2-mapping method to quantitatively differentiate edema from normal myocardium

**DOI:** 10.1186/1532-429X-11-S1-O4

**Published:** 2009-01-28

**Authors:** Shivraman S Giri, Yiu-Cho Chung, Ali Merchant, Tam Tran, Subha Raman, Orlando Simonetti

**Affiliations:** 1 The Ohio State University, Columbus, OH USA; 2 Siemens Medical Solutions, Columbus, OH USA

**Keywords:** Free Breathing, Myocardial Edema, Intensity Variability, Navigator Gating, Free Breathing Technique

## Introduction

T2-Weighted (T2W) imaging sequences can detect myocardial edema associated with acute inflammation, infarction, and the area at risk, but these techniques suffer from several drawbacks [1]. In this work, we describe a rapid technique for quantitative myocardial T2 mapping. This method is expected to quantitatively differentiate edema from normal tissue, to be insensitive to tissue motion, to easily distinguish edema from stagnant blood, and to be immune to surface coil sensitivity variations. The proposed T2-mapping method can be performed with either a short breath-hold or respiratory navigator gating.

## Purpose

To develop a rapid, quantitative method of myocardial T2-mapping to detect edema in patients with acute coronary syndrome.

## Methods

### Sequence

A single-shot T2-prepared SSFP acquisition was used to generate images with three T2-prep times: 0 (i.e., no T2 prep), 24, and 55 ms with parameters listed in Table 1. The technique is relatively motion insensitive due to the SSFP readout and the non-selective T2-preparation pulse. T2 maps were produced by fitting pixel intensities to a two-parameter mono-exponential model (Signal = M0 * exp(-TE/T2)), and setting any pixel with T2 > 120 ms to zero.

**Table 1:** Imaging parameters

Parameter	T2Prep SSFP	DB-TSE
TE (ms)	0, 24, 55	62
TR	2 × RR	2 × RR
Avg. FOV	350 × 400	350 × 400
Image Matrix	128 × 160	144 × 192
Flip angle	40	90

### Phantom

A two-compartment phantom was created to approximate the T1 and T2 values of normal (T1/T2 = 897/52.3 ms) and edematous myocardium (T1/T2 = 1119/101.8 ms) [2]. Phantom T1 and T2 values were verified using a standard spin echo sequence.

### In-vivo

T2 Maps were acquired in 9 healthy subjects to determine the normal range of T2 values. Three short-axis and two long-axis views were imaged during breath hold (duration ~5 HB) and in free breathing using navigator gating. In 5 subjects, four averages were acquired with navigator gating to test the benefits of increased SNR. Average T2 values were calculated in 16 myocardial segments using both methods and compared. Measurements were pooled to obtain global mean and standard deviation to investigate inter-subject and inter-segment variability.

### Signal intensity variability

In six healthy subjects, T2W images using conventional dark-blood STIR turbo spin echo (DB-STIR-TSE) were acquired and compared to the T2 maps generated using the proposed method. Only anterior coil elements were used to investigate signal variability due to surface coil intensity variation, as well as motion induced signal loss. Parameters are listed in Table 1. In each subject, average signal was computed and normalized to the maximum segment. The standard deviation (SD) of this normalized mean was used as a measure of variability.

### Animal studies

Three pigs underwent 90 minute LAD occlusion and were imaged with breath-hold within six hours of reperfusion.

## Results

### Phantom

T2 values were slightly overestimated (107.9 ms vs. 101.8 ms, and 60.7 ms vs. 52.3 ms) in the two phantom compartments.

### In-vivo

**Figure 1:**
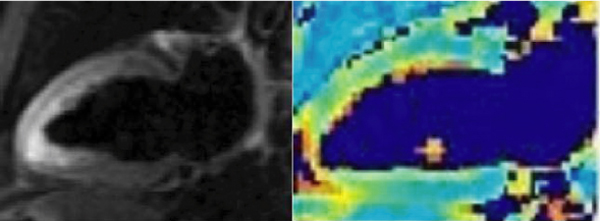
**T2 Weighted image (DB-STIR-TSE) (I) showing high signal intensity due to stagnant blood in the apical regions**. The corresponding T2 Map clearly distinguishes myocardium from stagnant blood.

T2 values did not show significant variation among the 16 segments (p = 0.277, ANOVA) or between breathhold and free breathing techniques (p = 0.76, paired t-test). The mean T2 and standard deviation were 51.54+3.5 ms (range: 49.6 to 53 ms). Figure 1 demonstrates the discrimination of static apical blood from myocardium in the T2 map.

### Signal intensity variability

**Figure 2:**
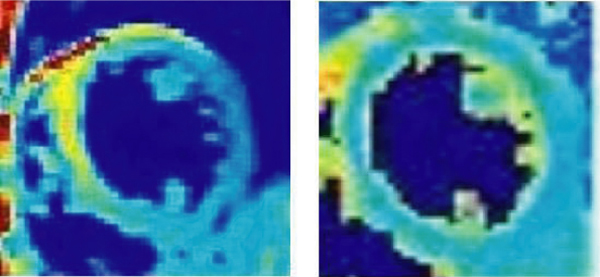
**T2W image (left) shows a lot of signal variability due to surface coil sensitivity variations and motion as compared to the corresponding T2 Map (right)**. Similar color map used in both images for comparison.

Signal in T2W DB-STIR-TSE showed high variability (36.7%) while T2 maps showed no such variation (3%) as seen in Figure 2.

### Animal study

**Figure 3:**
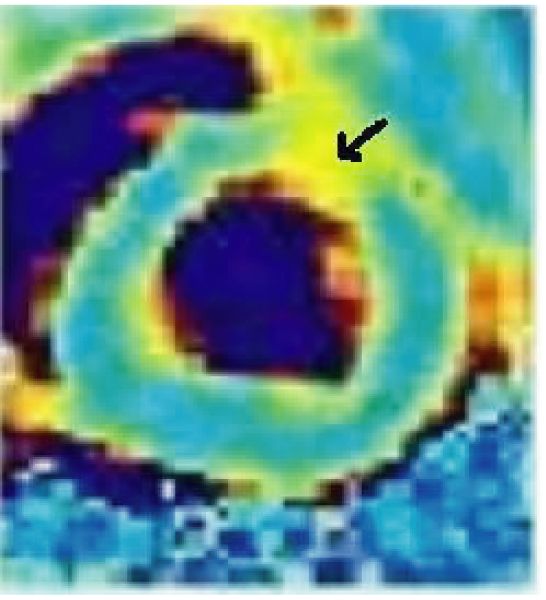
**T2 Maps from a pig showing enhanced T2 in anterior segment (arrow)**. In this pig, T2 in anterior segment was 83.5+11 ms vs. 58.6+4.5 ms in inferior segment

Results from the pig study are shown in Figure 3 and Table 2. In each animal, edematous segment showed a T2 value > 2 SD of remote segment.

**Table 2:** Results from pig studies.

Animal #	Infarcted segment	Remote normal segment
1	83.5,11	58.6,4.5
2	80.4,6.8	51.1,5.6
3	83.6,10	58.4,7.2

The values are mean, SD of T2 (ms) in mid ventricular SAX slice.

## Conclusion

We have demonstrated a rapid method of T2-mapping for quantitative detection of myocardial edema. Direct quantification of T2 eliminates many unwanted sources of signal variation, and removes the subjectivity of observer interpretation of bright regions. Further studies with patients are required to assess sensitivity and specificity.
